# A contemporary insight of metabolomics approach for COVID-19: Potential for novel therapeutic and diagnostic targets

**DOI:** 10.3126/nje.v10i4.33964

**Published:** 2020-12-31

**Authors:** Mohammad Asim, Brijesh Sathian, Indrajit Banerjee, Jared Robinson

**Affiliations:** 1 Surgery Department, Trauma Surgery, Hamad General Hospital, Doha, Qatar; 2 Geriatrics and long term care Department, Rumailah Hospital, Hamad Medical Corporation, Doha, Qatar; 3 Centre for Midwifery, Maternal and Perinatal Health, Bournemouth University, Bournemouth, England, United Kingdom; 4 Sir Seewoosagur Ramgoolam Medical College, Belle Rive, Mauritius

**Keywords:** COVID-19, metabolomics, severe disease, diagnosis, therapeutic targets

## Abstract

The Coronavirus disease 2019 (COVID-19) pandemic is caused by rapidly spreading pathogenic virus known as severe acute respiratory syndrome coronavirus 2 (SARS-CoV-2), that affects vast majority of population worldwide. Although, around 80% of the cases had mild infection but still remaining 20% had developed respiratory failure and dysfunction of other organs that necessitate urgent oxygen therapy or specific interventions. Therefore, it is imperative to establish novel prognostic approaches to screen patients at high-risk of developing severe complications. The primary focus of current research for COVID-19 is to discover safe and efficacious vaccine for prevention and effective treatment for better management of the patients to overcome the pandemic. To achieve this goal, it is imperative to have better understanding of the molecular pathways involved in the pathophysiology and progression of severe COVID-19. The surge for reliable diagnostics and therapeutics targets for COVID-19 highlighted the great potential of high-throughput approach like metabolomics which may enable the development of personalized medicine.

## Background

The Coronavirus disease 2019 (COVID-19) pandemic is caused by rapidly spreading pathogenic virus known as severe acute respiratory syndrome coronavirus 2 (SARS-CoV-2), that affects vast majority of population worldwide [1,2]. Although, around 80% of the cases had mild infection but still remaining 20% had developed respiratory failure and dysfunction of other organs that necessitate urgent oxygen therapy or specific interventions [3]. The complex process of the excessive production of inflammatory mediators and formation of microvascular thrombi resulted from endothelial activation and glycocalyx degradation that may result in severity of COVID-19 [4].

Therefore, it is imperative to establish novel prognostic approaches to screen patients at high- risk of developing severe complications. The primary focus of current research for COVID-19 is to discover safe and efficacious vaccine for prevention and effective treatment for better management of the patients to overcome the pandemic. To achieve this goal, it is imperative to have better understanding of the molecular pathways involved in the pathophysiology and progression of severe COVID-19 [5].

To date, various protein biomarkers have been identified that are potential candidates for early diagnosis and prognosis in COVID-19 patients [6]. However, molecular characterization of novel disease markers associated with COVID19 are crucial for improving the outcomes of COVID-19 patient. Thus, the surge for reliable diagnostics and therapeutics targets for COVID-19 highlighted the great potential of high-throughput approach like metabolomics which may enable the development of personalized medicine to target the dysregulated pathways, as revealed by the identified molecular profiles [7].

### Metabolomics approach for qualitative and quantitative analysis of endogenous metabolites in COVID-19 patients

**Figure 1:**
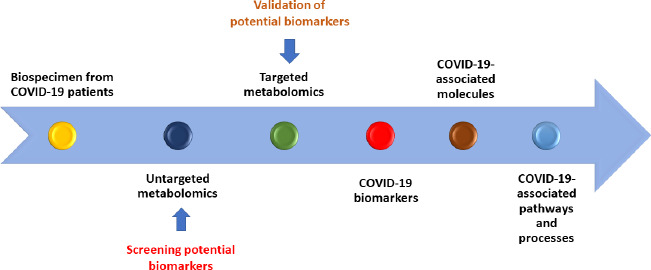
Metabolomic profiling in COVID-19 patients

Metabolomics approach provides quantitative analysis of the physiological metabolites such as amino acids, sugars, organic acids, lipids, acylcarnitines and several other small molecules present in the biospecimens (i.e. blood, urine, feces and saliva) of an individual [8]. Therefore, slight variation in the pattern and quality of these endogenous molecules can precisely indicate metabolic dysfunction secondary to pathogenic infection. These metabolites are the byproducts of genomic, proteomic, and environmental interactions, which may help in better understanding of the comprehensive chemical biomarkers of COVID-19 [9]. Owing to greater sensitivity and specificity, metabolomics could be used an ideal tool for understanding the association between the human body response and SARS-CoV-2 infection. The commonly used analytical techniques for research on metabolomics include liquid chromatography-mass spectrometry (LC-MS), gas chromatography-mass spectrometry (GC-MS) and nuclear magnetic resonance spectroscopy (NMR), [4]. However, the selection of an appropriate technique is guided by the sample matrix, quantity of the sample, concentration and properties of the metabolites of interest. Mass spectrometry is the primary technique used for metabolomics studies for clinical diagnostics which can detect and quantitate endogenous and exogenous metabolites that can be potentially used as therapeutic targets and diagnostic biomarkers. Metabolomic analysis could be based on either targeted and untargeted approach (Figure 1). The wide range detection of specific metabolites or groups of metabolites in a disease state or in physiological stimuli is referred as targeted metabolomics. This approach is based on detection and quantification of specific metabolites which are limited in number. On the other hand, untargeted approach simultaneously covers identification of a broader range of novel metabolites which may constitute distinct chemical as well as physical composition.

### Therapeutic implications of metabolomic profiling in COVID-19: Prediction of Antiviral Drug Efficacy

Study of interaction of SARS-CoV-2 infection with the host metabolome may help in developing therapeutics targets and assessment of their efficacy to treat COVID-19. The currently available antiviral medications like ribavirin, remdesivir, and favipiravir against SARS-CoV-2 which helps in lowering the viral load necessitates intracellular ATP-dependent activation to convert into active triphosphate forms which become functionally active [10]. Recent metabolomic studies among COVID-19 patients who developed acute respiratory distress demonstrated that the plasma metabolomic signatures of these patients are similar to that of sepsis syndrome [10,11]. Notably, sepsis patients had severe illness which results in poor bioenergetics profile, which resembles metabolomic signatures in COVID-19 patients manifested with suppression of metabolism, degranulation of platelet and impaired macrophage function [7]. The SARS-CoV-2 infection related with pathophysiology as identified by the metabolomic profiling of the COVID-19 patients and affects the efficacy of available anti-viral drugs as their functional activation is impaired due to low endogenous cellular energy of the infected individuals. The metabolomic studies have suggested that the deficiency of energy-rich cellular metabolites could affect the functionality of the potential anti-viral medication in suppressing the viral replication. Hence, metabolomic phenotyping may improve the therapeutic potential of replicase-transcriptase inhibitors (ATP-dependent) against COVID-19 which are at present being investigated under clinical trials [10]. Therefore, characterization and adjustment of the host bioenergetic state with a personalized approach may improve long-term outcomes of COVID-19 patients.

### Metabolomic analyses of lipids, amino acids and other molecules in COVID-19 patients

The human plasma mainly constitutes metabolites and lipid which upon severe illness, shows abnormal physical and chemical properties that may contribute to the pathophysiology of the disease. A study by Shen et al [11] demonstrated dysregulation in lipid related metabolites, of which the levels of eleven steroid hormones are elevated which are supposed to be associated with macrophage modulation. Another study suggested the possible involvement of two pathways such as metabolites of corticosterone synthesis and kynurenine pathway for NAD+ synthesis in COVID-19 response [12]. Contrarily, the lower levels of sphingolipids were identified which modulates activation and migration of macrophage to site of inflammation and apoptosis. Also, glycerophospholipids were lowered in COVID-19 patients who control platelet degranulation and phagocytosis; similarly, levels of choline and its derivatives, which regulate cytokine secretion, were decreased [11]. The metabolites involved in metabolism of amino acid and its derivatives were also decreased in COVID-19 patients which suggest hepatic dysfunction system as earlier study demonstrated interference of interferons induced by viral infection with the urea cycle [13]. Overall, the serum samples of patients with COVID-19 infection showed reduction in the levels of around 100 lipid molecules upon metabolomic profiling. Notably, dysregulation of lipidome may directly influence the cycle of coronavirus infection. Therefore, the reported variability in metabolome and lipidome has potential for the development of therapeutic targets for COVID-19.

Hierarchical clustering analysis show links between COVID-19, IL-6 levels, and amino acid metabolism, purines, acylcarnitines and fatty acids [7]. Metabolism pathways for five amino acids were found to be significantly affected by COVID-19 using metabolite set enrichment analysis of merged targeted and untargeted metabolomics data. COVID-19 patients with moderate-to-high levels of IL-6 show an elevation in creatine, creatinine, polyamines spermidine and acetyl-spermidine. Collectively, the observations indicate renal dysfunction which was supported by clinical quantitation of creatinine and blood urea nitrogen. The study further showed that in COVID-19 patients, there is hyperglycemia, elevation in glycolysis metabolic intermediates and pentose phosphate pathways. The metabolomic analysis corresponds to markers of inflammation and renal function that are clinically assessed in the laboratory [7]. Analysis of hydrophilic and hydrophobic metabolites from plasma of 34 COVID-19 patients led to the identification and quantification of 431 metabolites and 698 lipid molecules, revealing significant changes in the plasma metabolome and lipidome [14]. These findings demonstrated the association between abnormal metabolomic profile and disease severity. Therefore, a strong relationship has been observed between the metabolomic and lipidomic dysfunction indicating that the SARS-CoV-2 infection affects the complete metabolism [15].

### Metabolomics based diagnostic biomarkers for COVID-19

Despite the challenges involved in the selection of mass spectrometry method for the metabolomics and interpretation of enormous metabolomic data, there exist an immense potential for understanding the diagnostic and prognostic biomarkers of COVID-19. A recent study performed metabolomics on the serum samples obtained from severe and non-severe COVID-19 patients and analyzed serum from ill non-COVID patients and healthy individuals for comparison [11]. The authors reported marked variation in several metabolites namely carbohydrates, fatty acids, amino acids and glycerophospholipids. Another study also demonstrated difference in the chemical composition of plasma using metabolomics in COVID-19 cases with fatal, severe and mild disease as opposed to the healthy volunteers [14]. Although, these studies involved smaller number of participants, the variability in the metabolites and the associated outcomes based on severity of SARS-CoV-2 infection should be considered carefully due to paucity of information regarding COVID-19. Still these findings highlighted the presence of host response to infection; more precisely in terms of decrease in blood amino acids that seems to be a potential target for developing quick point-of-care screening tool. Therefore, development of specific assay based on metabolomics for COVID-19 diagnostic testing may provide a simple and effective method for mass screening.

In addition, to the possibility of metabolomics based diagnostic biomarkers, this approach could be equally beneficial for the development of the novel biomarkers for prognosis. It has been shown that various chemical changes in the blood could be identified days before the patients developed critical illness by metabolomic profiling [11]. Therefore, changes in the carbohydrates, amino acids, lipids, and other biomolecules in the blood of infected individuals may be used as potential metabolomic tool for diagnostic and prognostic testing which may complement the existing COVID-19 antigen and nucleic acid testing. Furthermore, a precision medicine-based approach is needed to develop a robust digital platform for predictive diagnostics and effective therapeutics for better outcomes.

### Expert Opinion

SARS-CoV-2 has spread at an excessive and unprecedented rate infecting million globally. Currently, the diagnostic and prognostic factors and the efficacy of antiviral drugs used to treat patients with COVID-19 has little available data. Moreover, the mass assessment of effective treatment on a large global scale is unrealistic as the dearth of information makes the establishment of a proven therapeutic regime near impossible. Furthermore, the diagnosis of COVID-19 has been plagued by numerous setbacks due to lack of sensitivity and specificity. It is therefore imperative to develop a simple and robust diagnostic test that may retrofitted onto the existing technologies. To overcome these challenges metabolomics approach may hold the solutions, as it can be applied for precision medicine to deliver personalized treatment regime for each individual patient thereby substantiating the efficacy of the treatment. This approach also enables rapid adjustment of treatment for any minute metabolic dysfunction that may possess major risk factor for development of critical illness. The uniqueness of metabolomics approach is that its application allows accurate diagnosis and optimized treatment that may save the lives of high-risk individuals. The role of metabolomics can no longer be renounced as it plays an important role in better understanding of the current pandemic and may have potential role to play in the future pandemics. The advantage of metabolomics is that it is currently established and has potential for personalized medicine. As with all the techniques and counter measures being explored for the SARS-CoV-2, more data and time will be required to establish its implementation and long-term applicability. It is evident that high-through technology such as metabolomics will be a major stake holder in future pandemics.

## Conclusion

Evidence suggest that the viral infection triggers novel pathways and changes the cellular metabolites and so several proteins and metabolite levels become abnormal after SARS-CoV-2 infection. Some of these signature metabolites can be used as therapeutic targets to revive normal levels in order to mitigate the effects of COVID-19. While, abnormal levels of other metabolites can be utilized as screening markers against COVID-19. Therefore, identification of potential diagnostic and therapeutic targets using metabolomics is a significant step toward future predictive diagnostics and drug discovery to combat infectious diseases. Advanced molecular technology and bioinformatics analysis of metabolomic data may help to develop therapeutic targets and better understanding of the disease pathophysiology of COVID-19. Therefore, an individualized medicine-based approach is needed to develop a robust digital platform using high-throughput technology for predictive diagnostics and effective therapeutics for better outcomes. Moreover, there is an urgent need to improve the process of diagnostic and therapeutic approaches by integrating metabolomics into clinical practice and develop high-through database for precision medicine.
